# Cowpea seeds from plants subjected to restricted-and full-irrigation regimes show differential phytochemical activity

**DOI:** 10.1186/s40529-022-00360-x

**Published:** 2022-10-12

**Authors:** MirReza Miri, Farshad Ghooshchi, Hamid Reza Tohidi Moghadam, HamidReza Larijani, Pourang Kasraie

**Affiliations:** Department of Agriculture, Varamin-Pishva Branch, Islamic Azad University, Varamin, Iran

**Keywords:** Drought stress, Legumes, Mineral nutrition, Phytochemicals, *Vigna unguiculata* L. Walp

## Abstract

**Background:**

Water scarcity is responsible for losses in the yield of many plants and this is expected to continue due to climate change. However, cowpea which is known for its drought tolerance, is considered as a plant without limitations to climate change. A two-year experiment was conducted to evaluate the effect of water restriction on phenolic compounds, antioxidant capacity and leaf nutrients concentration of four cultivars of cowpea at different growth stages. At second leaf stage, two irrigation regimes were initiated (Water irrigation was applied after 75% and 55% of field capacity, as well watered and drought stress treatment, respectively).Plants samples were collectedat three stages(immature pod, immature seed and dry seed stage) for total phenol and flavonoids content, ortho-diphenols andantioxidant capacity measurement and leaves sampling for nutrients concentration.

**Results:**

The results indicated that polyphenolic compounds and antioxidant capacity increased under drought conditions. However, in both irrigation regimes, immature pods had the higher polyphenolic compounds, antioxidant capacity and leaf nutrients concentration rather than immature seeds and dry seeds. Among the genotypes, ILC482 revealed the highest content of total phenolics and ortho-diphenols (6.9 and 3.57 mg GA g^−1^dry weight, respectively). In addition, nitrogen, phosphorus and magnesium concentration of leaves were higher in ILC482 genotype. Under drought stress, ILC482 maintained higher ABTS radical scavenging capacity (0.0083 mmol Trolox g^−1^dry weight) compared to other genotypes.

**Conclusions:**

It is suggested that drought stress affect the quality of cowpea productions through polyphenolic compounds, ABTS and DPPH radical scavenging capacity which can be used as a helpful strategy to save water in the regions where water is scare.

## Introduction

Drought stress is one of the most critical environmental conditions which affect crop production and yield, especially in regions where there is water scarcity. Cowpea (*Vinga unguiculate* L. Walp) is considered a drought tolerant plant grown by subsistence farmers in semi-arid regions which experience frequent heat and drought during the growing season. Indeed the ability to survive high-temperature conditions without irrigation system and with low rainfall makes cowpea an important legume in the arid and semi-arid regions (Miri et al. 2021). Cowpea is cultivated for its usage as green manure, and food grain for human and animal feed. It is a key crop in food security where the green pods, fresh and dry seeds can be consumed (Patel et al. 2019).

Interestingly, water deficit during the vegetative period as opposed to the flowering or fruit forming stages has less impact on seed yield (Nassourou et al. 2016).Water deficit changes the composition of plant yield, enhancing nutrients, total fiber and protein content. Moreover, stress conditions induce photooxidative stress, which enhance the synthesis of bioactive and secondary metabolites.Cowpea seeds have diverse group of polyphenols which are in charge of antioxidant properties. The polyphenols neutralizes the adverse effect of reactive oxygen species (ROS) which occur in unfavorable environmental conditions (Sadeghipour 2020; Keshavarz and Khodabin 2019). Daryanto et al. (2015) and Moreira-Araújo et al. (2018) reported that water deficit increases total polyphenols and flavonoids in legume production seeds.

However, under climate change, extreme unfavorable conditions such as drought and heat stress affect cowpea production in the arid and semi-arid regions, leading to yield loss(Venkatesh and Park 2014).This pattern is expected to continue as cowpea is the main grain legume of Mediterranean areas, Africa tropics and sub-tropics (Prgomet et al. 2019). In addition, Europe is facing a deficit of about 70% of grain legumes (Santos et al. 2020) and 60% of the current legumes producing areas in Sub Saharan Africa are projected to become unsuitable for common bean production before the end of the century (Carvalho et al. 2019; Karapanos et al. 2017; Keshavarz and Sadegh-Ghol-Moghadam 2017).

Further more, water stress affects the composition of cowpea seeds and improves the free amino acid pool but reduces their incorporation in the protein chain, this however, depends on the duration and intensity of the stress, crop growth stage and genotypic variations (Keshavarz 2020; Sadeghipour 2020). In addition, the optimization of water management during cowpea cultivation is required to improve the yield quality and reduce loss. For example, Ceritoglu and Erman (2020) reported that drought stress at vegetative and reproductive stages resulted in approximately 15% and 30% yield reduction respectively, while drought stress during vegetative and flowering periods reduce grain quality and quantity of cowpea genotypes. It is therefore important to study the pod and seed chemical composition of cowpea as influenced by environmental conditions (such as drought stress), stage of growth and cultivar (Karami et al. 2016).

The application of restricted irrigation during plant growth is conducted mainly to evaluate seed composition, phenolic compounds and secondary metabolites. In this study, we examined the impact of restricted-and full-irrigation regimes on four cowpea genotypes, by characterizing the total phenolics, ortho-diphenols, flavonoids The 1,1-diphenyl-2-picrylhydrazyl (DPPH)and 2,2′-azino-bis(3-ethylbenzothiazoline-6-sulfonate) (ABTS) radical scavenging capacity and the nitrogen (N), phosphorus (P), potassium (K), calcium (Ca) and magnesium (Mg) content of the leaf at three growth stage. We envisage that this work would provide insight into the mechanisms of drought acclimatization in cowpea and further provide motivation for breeding cowpea genotypes to improve yield and consumption.

## Materials and methods

### Experimental detail

**Fig. 1:**
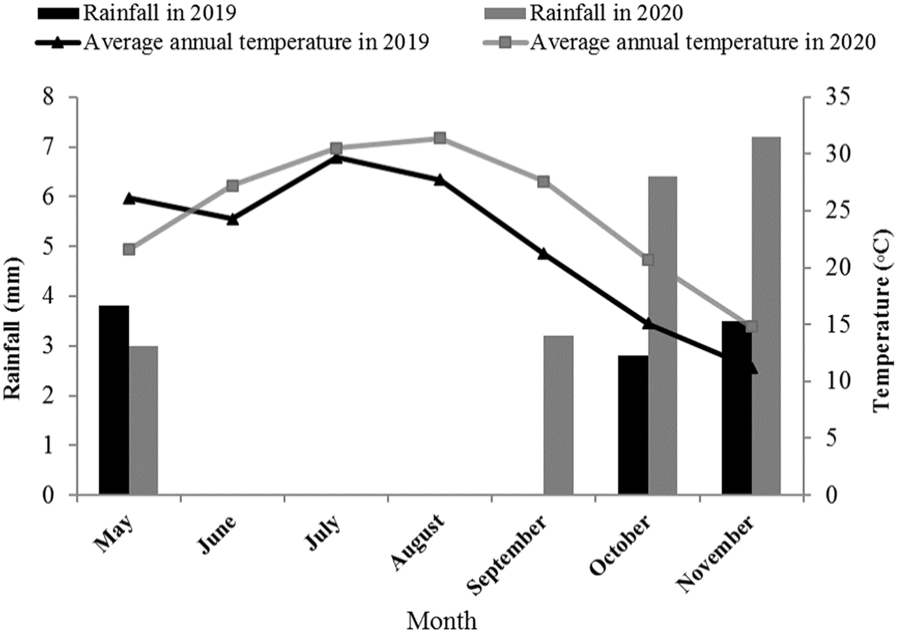
The average temperature and the total rainfall in the months of the growth season

The field experiment was conducted using a randomized complete block design arranged in split plot factorial with 3 replicates at the research farm of Islamic Azad University (35° 7′ to 35° 39′ N and 51° 26′ to 51° 55′ E),Tehran, Iran, during the growth period of 2019 and 2020, from June to August in both years (Fig. 1). The soil of the experimental site was sandy loam, with the neutral pH, having an organic matter (0.068%), P_2_O_5_ (2.89 kg ha^−1^) and 16% of moisture at permanent wilting point.

The cowpea cultivars (Arman, Azad, Adel and ILC482) were obtained from Seed and Plant Improvement Institute, Karaj, Iran. Cowpea cultivars were selected based on previous research and were previously submitted to laboratory for procedures of overcoming dormancy. Each of the Cowpea cultivars were grown in a six-row plot of 2.5 m × 4 m and spaced at 50 cm and 10 cm between and within rows (A 2 m space was between main block). Three pipes (with dripping spacing of 10 cm) were installed in the middle of each plot.

After 30 days of complete emergence (second definitive leaf -phenological stage V3), the irrigation regimes (irrigated and water shortage) was initiated. The control plot, (well-watered treatment) continued to receive daily irrigation (75% field capacity), and those on the drought stress treatment had their irrigation at 55% field capacity. From that moment, irrigation was re-established, until maintaining the humidity close to the field capacity following a methodology described by Keshavarz Mirzamohammadi et al. (2021a).For this purpose, the soil water content and the amount of irrigation water was calculated with a time domain reflectometer and the plots were irrigated to field capacity. For all the plots, the local recommended basal doses of chemical fertilizers were applied homogeneously into the soil at the rate of 50 kg ha^−1^, 90 kg ha^−1^ and 60 kg ha^−1^ as urea, single superphosphate and potassium sulfate, respectively.

### Data collection

Plants were harvested as immature pod, immature seed and dry seed stage to examine the effect of drought on phytochemical contents and antioxidant capacity of each sample. Based on Krizek et al. (1993) and the Folin-Ciocalteu methods (Temraz and El-Tantawy 2008) the contents of the total phenolic and flavonoids were tested in immature pod, immature seed and dry seed of cowpea. Determination of the ortho-diphenols content was done using High-performance liquid chromatography (HPLC) by Gallic acid as standard according to Bakhtiar et al. (2014).The DPPH and ABTS free radical scavenging capacity were measured using the method of Emami Bistgani et al. (2018).

Leaves samples were also harvested (at the three-developmental stage) to access leaf nutrients content. For this purpose, the leaves were harvested, washed in distilled water and then oven dried at (65 °C, 48 h) to constant weight. After grinding in a ball mill, all the dry mass was milled in a Wiley type mill, homogenized and stored for the determination of macronutrients content.The N concentrations were determined by the Kjeldahl method (sulfuric acid digestion extract). The P and K contents were determined by colorimetry (nitricperchloric acid digestion) and flame photometry, respectively. Finally, Ca and Mg were measured by atomic absorption spectrophotometry.

### Statistical analysis

The variance of two years of data, was analyzed using ANOVA and LSD’s mean wise comparison test (honest significant difference) at 95% significance level. SAS 9.1 and S-PLUS version 6.1 software, USA were used for Principal Component Analysis (PCA) based on biplot and clustering analysis.

## Results

### Analysis of variance

The analysis of variance showed that (Table 1) the main effect on each year was significant on content of total phenolics, content of flavonoids, ABTS radical scavenging capacity and seed Mg content. Except the flavonoids content and seed Ca content, other measured traits were significantly (*P* ≤ 0.01) affected by irrigation regimes. The two-way interaction of the years by irrigation showed a significant effect on content of ortho-diphenols. The effect between Cowpea cultivars was also significant on all traits except on the seed with Ca content. Accordingly, the content of ortho-diphenols, flavonoids, ABTS radical scavenging capacity, DPPH radical scavenging capacity, leaf N, P, K and Mg were significantly affected by the simple effect at developmental stage. The interaction of irrigation and Cowpea cultivars was also significant on ABTS radical scavenging capacity. Except the content of total phenolics and seed Ca content, all other traits were significantly affected by two-way interaction of irrigation at the developmental stage. Moreover, the interaction effect of Cowpea cultivars and respective year was significant on the flavonoids content, ABTS radical scavenging capacity and leaf K content. All other two-way and three-way interactions were not significant (Table 1).

**Table 1:** Combined analysis of variance on some physiological and agronomic traits and of the cowpea as affected by irrigation regimes, genotypes and development stage

SOV	df	Content of total phenolics	Content of ortho-diphenols	Content of flavonoids	ABTS radical scavenging capacity	DPPH radical scavenging capacity	Leaf N	Leaf P	Leaf K	Leaf Mg	Leaf Ca
Year	1	28.56*	1.14^ns^	1.10*	0.00*	0.00^ns^	2196^ns^	47.20^ns^	970^ns^	1.91*	0.58^ns^
Block (Year)	4	1.48	0.49	0.05	0.00	0.00	1166	57.07	134.90	0.16	0.14
Irrigation (IR)	1	124.10**	147.17**	1.89^ns^	0.00**	0.00*	51,551**	6076**	43,260**	23.30**	0.02^ns^
Year × IR	1	13.02^ns^	4.99*	0.11^ns^	0.00^ns^	0.00^ns^	640^ ns^	15.10^ns^	231^ns^	0.54^ns^	0.34^ns^
IR × Block (Year)	4	3.62	0.50	0.34	0.00	0.00	946	44.60	523	0.29	0.66
Genotype (G)	3	10.80**	4.32**	1.40**	0.00**	0.00**	4151**	252.60**	2454**	0.65**	0.00^ns^
Development stage (D)	2	1.45^ns^	2.09**	0.50**	0.00**	0.00**	1403**	135.09**	1252**	0.63**	0.04^ns^
IR × G	3	0.00^ns^	0.30^ns^	0.04^ns^	0.00**	0.00^ns^	64.3^ns^	9.52^ns^	24.90^ns^	0.00^ns^	0.04^ns^
IR × D	2	0.83^ns^	1.96**	0.47**	0.00**	0.00^*^	623**	84.90**	1371**	0.46**	0.06^ns^
G × D	6	0.07^ns^	0.02^ns^	0.00^ns^	0.00^ns^	0.00^ns^	16.08^ns^	1.28^ns^	16.8^ns^	0.00^ns^	0.00^ns^
IR × G × D	6	0.07^ns^	0.04^ns^	0.01^ns^	0.00^ns^	0.00^ns^	39.07^ns^	7.88^ns^	9.10^ns^	0.00^ns^	0.00^ns^
G × Year	3	0.24^ns^	0.18^ns^	0.10*	0.00*	0.00^ns^	10.37^ns^	4.09^ns^	319**	0.00^ns^	0.01^ns^
D × Year	2	0.08^ns^	0.06^ns^	0.09^ns^	0.00^ns^	0.00^ns^	3.08^ns^	1.11^ns^	0.61^ns^	0.00^ns^	0.02^ns^
IR × G × Year	3	0.37^ns^	0.00^ns^	0.00^ns^	0.00^ns^	0.00^ns^	71.98^ns^	1.98^ns^	9.39^ns^	0.00^ns^	0.00^ns^
IR × D × Year	2	0.21^ns^	0.04^ns^	0.10^ns^	0.00^ns^	0.00^ns^	104.2^ns^	3.90^ns^	3.35^ns^	0.01^ns^	0.02^ns^
G × D × Year	6	0.77^ns^	0.07^ns^	0.01^ns^	0.00^ns^	0.00^ns^	60.9^ns^	0.11^ns^	7.92^ns^	0.00^ns^	0.01^ns^
IR × G × D × Year	6	0.24^ns^	0.08^ns^	0.03^ns^	0.00^ns^	0.00^ns^	78.3^ns^	0.93^ns^	47.10^ns^	0.00^ns^	0.00^ns^
Error	88	0.47	0.11	0.03	0.00	0.00	48.06	3.56	64.10	0.02	0.07
CV (%)		10.90	10.59	7.54	5.97	8.35	6.25	4.34	7.05	9.11	11.80

*S.O.V.* The Source of Variation, *ns* not significant; * and ** significant at the 5% and 1% levels of probability, respectively

**Fig. 2:**
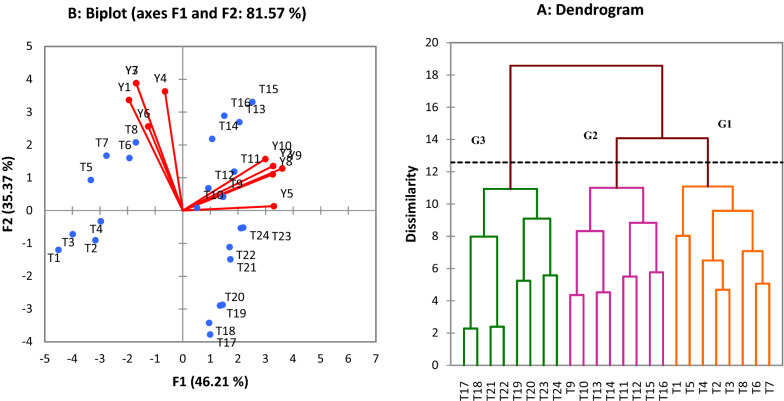
The results of dendrogram based on cluster analysis (**A**) and biplot of first and second components based on principal component analysis (**B**). Y_1_: Content of total phenolics, Y_2_: Content of ortho-diphenols, Y_3_: Content of flavonoids, Y_4_: ABTS radical scavenging capacity, Y_5_: DPPH radical scavenging capacity, Y_6_: Leaf N content, Y_7_: Leaf P content, Y_8_: Leaf K content, Y_9_: Leaf Mg content, Y_10_: Leaf Ca content, T_1_: Well-watered + Immature pod + Arman, T_2_: Well-watered + Immature pod + Azad, T_3_: Well-watered + Immature pod + Adel, T_4_: Well-watered + Immature pod + ILC482, T_5_: Well-watered + Immature seed + Arman, T_6_: Well-watered + Immature seed + Azad, T_7_: Well-watered + Immature seed + Adel, T_8_: Well-watered + Immature seed + ILC482, T_9_: Well-watered + dry seed + Arman, T_10_: Well-watered + dry seed + Azad, T_11_: Well-watered + dry seed + Adel, T_12_: Well-watered + dry seed + ILC482, T_13_: Drought stress + Immature pod + Arman, T_14_: Drought stress + Immature pod + Azad, T_15_: Drought stress + Immature pod + Adel, T_16_: Drought stress + Immature pod + ILC482, T_17_: Drought stress + Immature seed + Arman, T_18_: Drought stress + Immature seed + Azad, T_19_: Drought stress + Immature seed + Adel, T_20_: Drought stress + Immature seed + ILC482, T_21_: Drought stress + dry seed + Arman, T_22_: Drought stress + dry seed + Azad, T_23_: Drought stress + dry seed + Adel, T_24_: Drought stress + dry seed + ILC482

Cluster analysis (Fig. 2A) divided all studied treatments in the experiment into three separate groups based on all measured traits. Based on the dendrogram obtained from cluster analysis, it was observed that 8 treatments in the first group (T1, T5, T4, T2, T3, T8, T6 and T7), 8 treatments in the second group (T9, T10, T13, T14, T11, T12, T15 and T16) and 8 treatments were placed in the third group (T17, T18, T21, T22, T19, T20, T23 and T24). The results of these principal component analysis (Fig. 2B) showed that three components had an eigenvalue higher than one and were considered as effective components. The first and second components with 46.21% and 35.37%, respectively, had the highest relative variance and in total had 81.58% of the total variance. Biplot results obtained from the first and second components showed that T5, T7, T6 and T8 treatments were highly correlated with Y3, Y1, Y6 and Y4 traits. Also, other studied traits showed strong correlation with T14, T16, T13, T15, T9, T10, T11 and T12 treatments.

### Content of total phenolics

**Fig. 3:**
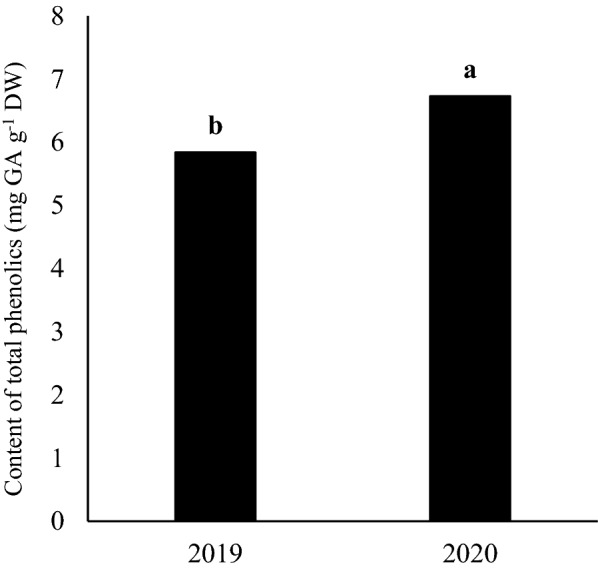
Main effect of year on content of total phenolics

**Fig. 4:**
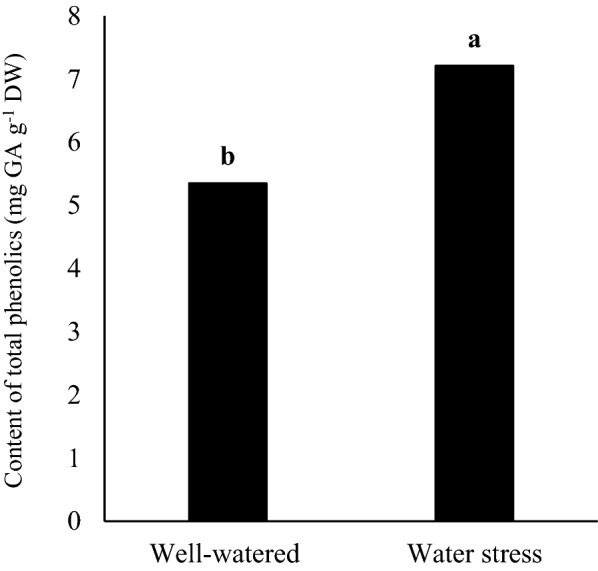
Main effect of irrigation levels on content of total phenolics

The results of ANOVA indicated that the main effect on each year was statistically significant for the content of total phenolics (Table 1). Accordingly, the maximum values of total phenolics content was obtained in the second year (6.73 mg GA g^−1^ DW), which was higher than first year by 13.2% (Fig. 3). Keshavarz Mirza Mohammadi et al. (2021b) observed that the production of phenolic compounds in the peppermint had little variation throughout the year, despite significant differences in air temperature were observed in different months. Results demonstrated that drought stress treatment decreased the content of total phenolics than well-watered (control) treatment by 25.7% (Fig. 4). Based on mean comparison results (Table2), total phenolics content responded differently to various genotypes treatments. The maximum total phenolics content belonged to ILC482 genotype (6.94 mg GA g^−1^ DW), which was higher than Arman, Azad and Adel by 18.7%, 7.06% and 12.1%, respectively (Table2). However, there were similarity between Azad and ILC482 genotypes, Azad and Adel genotypes and Adel and Arman genotypes.

**Table 2:** Main effect of genotype treatments on some physiological traits and leaf nutrients content of cowpea

Genotype	Content of total phenolics	Content of ortho-diphenols	DPPH radical scavenging capacity		Leaf N	Leaf P	Leaf Mg
	(mg GA g^−1^ DW)		(mmol Trolox g^−1^ DW)		(mg plant^−1^)		
Arman	5.64c	2.77c	0.02c		98.6b	40.2b	1.66b
Azad	6.45ab	3.34ab	0.03ab		114.6ab	43.5ab	1.83ab
Adel	6.10bc	2.95bc	0.03bc		106.3ab	43.2ab	1.80ab
ILC482	6.90a	3.57a	0.03a		123.6a	46.7a	1.99a

Each parameter with the same letter is not significantly different according to LSD test at the 5% level of probability

### Content of ortho-diphenols

**Fig. 5:**
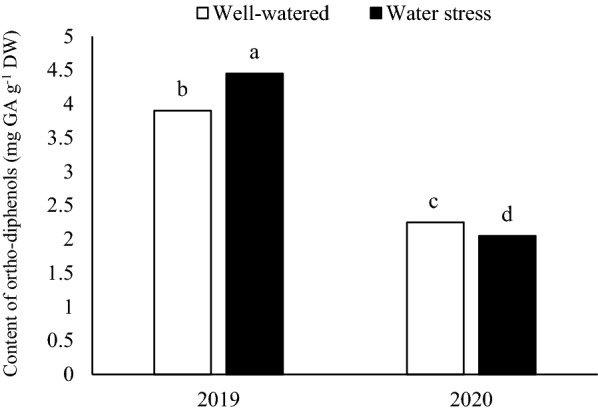
Two-way interaction of year × irrigation regime on content of ortho-diphenols

The results of combined ANOVA showed that a two-way interaction between each year and irrigation was statistically significant for the content of ortho-diphenols. When averaged across the year and irrigation treatments, the highest content of ortho-diphenols was obtained by those plants which were sown in first year and irrigated with normal irrigation (4.45 mg GA g^−1^ DW, Fig. 5), while content of ortho-diphenols decreased in the second year. Based on mean comparison results, various genotypes responded differently in their ortho-diphenols content. The highest value of ortho-diphenols was obtained from ILC482 genotype (3.57 mg GA g^−1^ DW) which was higher than the Azad, Adel and Arman genotypes by 6.44%, 17% and 22.4%, respectively (Table 2). The two-way interaction of irrigation regime × development stage (Table 3), indicated that the highest value of ortho-diphenols was achieved under drought stress and immature pods (4.51 mg GA g^−1^ DW). The lowest value of ortho-diphenols was observed at drought stress in immature seeds (1.84 mg GA g^−1^ DW) (Table 3).

**Table 3:** Two-way interaction of irrigation regime × development stage on some physiological traits and leaf nutrients content of cowpea

Irrigation regimes	Development stage	Content of ortho-diphenols	Content of flavonoids	ABTS radical scavenging capacity	DPPH radical scavenging capacity	Leaf N	Leaf P	Leaf K	Mg
		(mg GA g^−1^)	(mg CAT g^−1^ DW)	(mmol Trolox g^−1^ DW)	(mmol Trolox g^−1^ DW)	(mg plant^−1^)			
	Immature pods	2.25c	2.45c	0.00d	0.02c	136.8a	52.4a	138.6a	2.39a
Well-watered	Immature seeds	1.84d	2.16d	0.00e	0.02d	126.4b	49.06b	128.8b	2.18b
	Dry seeds	2.37c	2.49bc	0.00d	0.02c	125.9b	48.3b	124.9b	2.11b
	Immature pods	4.51a	2.7a	0.00a	0.03a	95.2c	37.8c	98.2c	1.49c
Drought stress	Immature seeds	4.09b	2.58b	0.00b	0.03b	84.0d	34.5d	87.6d	1.24d
	Dry seeds	3.92b	2.5bc	0.00c	0.03b	96.4c	38.4c	102.6c	1.53c

Each parameter with the same letter is not significantly different according to LSD test at the 5% level of probability

### Content of flavonoids

Two-way interactions of irrigation regimes × developmental stage was significant for flavonoids content (Table 1). When averaged across irrigation regimes and development stage treatments, the highest flavonoids value (2.7 mg CAT g^−1^ DW) was obtained by those plants which were sown in irrigated plot and at immature pods stage, while flavonoids value decreased in drought condition by 9.25%, 16% and 0.4% in immature pods, immature seeds and dry seeds stagees respectively compared to well-watered condition (Table 3).However, there was no statistical difference between immature pods stage in well-watered and drought condition.

The two-way interaction between irrigation × genotype and genotype × year were significant for flavonoids (Table 1). In each growing season, the maximum flavonoids content was in ILC482 genotype (2.83 and 2.51 mg CAT g^−1^ DW, for the second and first year, respectively); although there were no significant differences between Arman genotype (Table 4) in both years. The lowest flavonoids content was observed in the first year as (2.18 mg CAT g^−1^ DW) and second year as (2.27 mg CAT g^−1^ DW) (Table 4). Moreover, by average of all genotype in each year, flavonoids content rose by 6.8% in the second year in comparison to the first year.

**Table 4:** Two-way interaction year × genotype treatments on content of flavonoids, ABTS radical scavenging capacity and leaf K content

	Content of flavonoids		ABTS radical scavenging capacity		Leaf K	
Genotype	(mg CAT g^−1^ DW)		(mmol Trolox g^−1^ DW)		(mg plant^−1^)	
	2019	2020	2019	2020	2019	2020
Arman	2.18f	2.27ef	0.00g	0.00f	103.9d	103.4d
Azad	2.49de	2.69b	0.00de	0.00b	109.9c	119.3b
Adel	2.38de	2.47cd	0.00ef	0.00cd	111.6c	112.2c
ILC482	2.51c	2.83a	0.00c	0.00a	118.1b	129.3a

Each parameter with the same letter is not significantly different according to LSD test at the 5% level of probability

### ABTS radical scavenging capacity

**Fig. 6:**
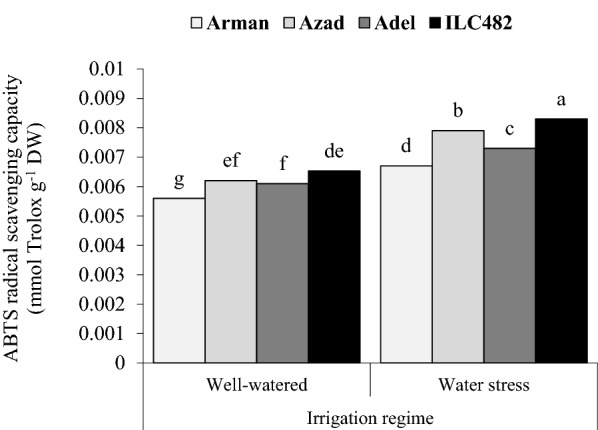
Two-way interaction of irrigation regime × genotype on ABTS radical scavenging capacity

The ABTS radical scavenging capacity was significantly affected by the two-way interaction of irrigation × genotype, irrigation × development stage and genotypes × year (Table 1). The average ABTS radical scavenging capacity of cowpea genotypes in drought stress treatment and immature pods was 0.0081 (mmol Trolox g^−1^ DW), while the amount of this trait for cowpea plants grown in well-watered condition and same development stage was 0.0063 (mmol Trolox g^−1^ DW) which was lower by 22.2% (Table 3). In stressed plants, sampling at immature seeds and dry seeds caused a lower ABTS radical scavenging capacity (with average of 0.0074 and 0.0070 mmol Trolox g^−1^ DW), however, in well-watered condition and immature seeds, ABTS radical scavenging capacity decreased but in dry seeds stage it significantly increased again (Table 3).The averaged ABTS radical scavenging capacity of cowpea genotypes in first year was 0.0065 (mmol Trolox g^−1^ DW), while the amounts of this trait for cowpea plants grown on second year was 0.0070 (mmol Trolox g^−1^ DW) which was higher by 7.42% (Table 4). The highest capacity of ABTS radical scavenging capacity (0.0078 kgha^−1^) was in second year and ILC482 genotype which was higher than Arman, Azad and Adel by 19.23%, 5.12% and 12.8%, respectively. Mean comparison results revealed that ABTS radical scavenging capacity (averaged of genotype treatments) was 0.0075 (mmol Trolox g^−1^ DW) under drought stress condition and well-watered treatment decreased ABTS radical scavenging capacity up to 18.6% (Fig. 6).The highest ABTS radical scavenging capacity (0.0083 mmol Trolox g^−1^ DW) was recorded in ILC482 genotype and drought treatment decreased by 19.2%, 4.81% and 12.4% when compared to Arman, Azad and Adel genotype (Fig. 6) respectively.

### DPPH radical scavenging capacity

The results showed that the DPPH radical scavenging capacity responded differently to the cowpea genotypes treatment (Table 2).The ILC482 genotype had the highest DPPH radical scavenging capacity (0.036 mmol Trolox g^−1^ DW), while the lowest DPPH radical scavenging capacity belonged to the Arman genotype (0.028 mmol Trolox g^−1^ DW) (Table 2). The capacity of DPPH radical scavenging was significantly affected by the two-way interaction of irrigation × development stage and simple effect of genotype (Table 1).When compared with well-watered treatment (at average of developmental stage), the DPPH radical scavenging capacity of plants grown in drought condition significantly increased by 27.7% (Table 3).The results obtained from the mean comparison illustrated that the cowpea plants in immature pods developmental stage grown in drought stress condition had the highest DPPH radical scavenging capacity with an average of 0.038 (mmol Trolox g^−1^ DW) and increased by 7.89% as compared to other developmental stages (Table 3). However, in drought condition, DPPH radical scavenging capacity of the dry seeds increased by 3.5% and 10.7% in immature pods and immature seeds, respectively (Table 3).

### Leaf N content

The genotypes significantly affected the leaf N content and leaf area was lower in Arman genotypes than the other genotypes (Table 2). However, the differences were not statistically significant between Azad, Adel and ILC482 genotypes (Table 2).Across the irrigation regimes and development stage, well-watered plants in immature pods stage had significantly highest leaf N content while the lowest leaf N content was achieved in drought stress and immature pods stage (Table 3). In well-watered conditions, leaf N content gradually increased in immature pods and immature seeds stage by 7.9% and 0.39% compared to dry seed stage (Table 3). However, leaf N content decreased by 1.24% in immature pods and 12.8% in immature seed compared to dry seed stage under drought condition.

### Leaf P content

Among genotype treatments, ILC482 and Aman genotypes showed the maximum and minimum leaf P content by average of 46.7 and 40.2 (Table 2).When averaged by irrigation regimes (Table 3), the highest leaf content was obtained by those plants which were harvested at immature pods (45.1 mg plant^−1^), while leaf P content decreased under drought conditions by 26.08% compared to well-watered conditions (averaged by development stage).

### Leaf K content

In terms of leaf K content, various developmental stage responded differently to irrigation levels (Table 2). The maximum leaf K content belong to well-watered stage and immature pods (138.6 mg plant^−1^). However, the leaf K content was impacted by drought stress. Averaged by developmental stage, drought treatment had lower leaf K content by 26.48% compared to well-watered situation. Generally, by a two years average (Table 4), ILC482 genotype had the highest leaf K content (123.7 mg plant^−1^), while, Arman genotype had the lowest leaf K content (103.6 mg plant^−1^).

### Leaf Mg content

**Fig. 7:**
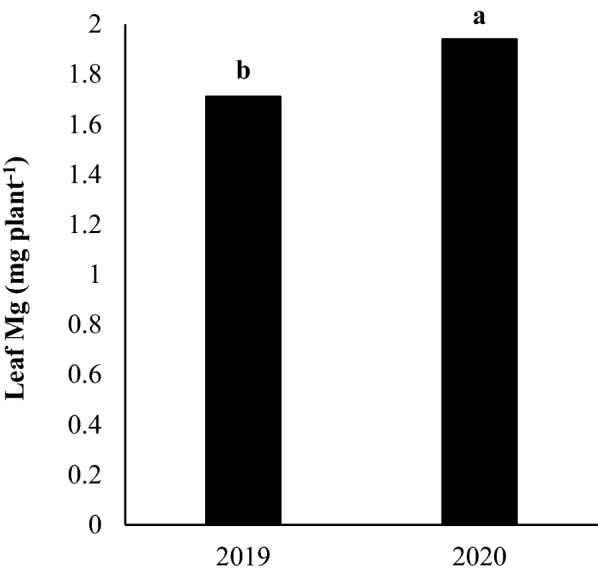
The effect of year on content of leaf Mg

Leaf Mg content was found to be higher in the second year (1.94 mg plant^−1^) compared to the first year, which might be attributed to better growing conditions in the first year (Fig. 7). Also, leaf Mg content responded differently to genotypes treatments such that the highest leaf Mg content was obtained by the ILC482 genotype with an average of 1.99 mg plant^−1^while the Arman genotype had the lowest contents of leaf Mg with the averages of 1.66 mg plant^−1^ (Table 2). When averaged across development stage treatments, the drought stress caused a 36.03% decrease in leaf Mg content. The obtained results from the mean comparison showed that the cowpea plants grown under well-watered conditions had different response to development stage, with the highest leaf Mg content (2.39 mg palnt^−1^) observed in immature pods grown under well-watered conditions but decreased by 8.0% and 16.2% in immature seeds and dry seeds, respectively (Table 3). By contrast, the lowest contents of leaf Mg belongs to immature seeds stage and increased by 16.7% and 18.9% in immature pods and dry seeds respectively.

## Discussion

### Phenolic compounds

Plant bioactive compounds, such as total phenolics, flavonoids ortho-diphenols play important roles in the plant development and human diet. It was revealed that higher content of total phenolic contents and lower chlorophyll content (some data not shown) were found in plants under water stress. Our results are in agreement with the findings that in stress conditions and when plants loss their chlorophyll, phenolic compounds increase and act as photoprotectors limiting light influx to the photosynthetic reaction center due to absorption of light before penetration into the mesophyll cells (Keshavarz et al. 2018; Shinohara et al. 2011) which lead to lower chlorophyll degradation.

However, physiological function of phenolic compounds as free radicals scavenging should also be considered here. The generation of ROS in oxygen evolving complex is intensified by water scarcity condition, as light value is optimal for photosynthesis II center in normal irrigation plants. This became extremely higher in stressed plants (Rah Khosravani et al. 2017). The exposure of the photosynthesis apparatus to high excitement energy, could enhance production of free radicals. Total phenolics, ortho-diphenols and flavonoids content help plants to survive through stress conditions due to reduction in oxidative damage in cell, by reducing the levels of free radicals. Moreover, phenolic compounds increased in drought stress might be due to over-expression of l-phenylalanine ammonia-lyase (PAL) genes in drought conditions for elimination of oxidative stress (Albergaria et al. 2020). Averagely, by developmental stage, increasing irrigation water significantly decreased phenolic compounds in cowpea plants, probably due to the role of these molecules in free radical scavengers and their redox properties which reduced hydrogen donors and singlet oxygen quenchers under drought conditions. In Fig. 5, ortho-diphenols strongly changed by year and irrigation supply. Indeed, when plants were subjected to drought stress, the content of ortho-diphenols increased which translated to lignin formation and structural integrity and resulted to higher drought resistance. The content of ortho-diphenols and flavonoids were higher in immature pods and gradually decreased with the crop maturity (averaged by irrigation regimes) in both years, indicating that in this stage cowpeas are a rich source of these secondary metabolites. These results are in agreement with Weidner et al. (2018) where they achieved the highest content of polyphenolic content in early harvested cowpea. Among the genotypes, ILC482 had the highest total phenolic content, ortho-diphenols and flavonoids, but genetic and environment factors might have accounted for the slight variation between genotypes.

### Antioxidant activity

Environmental stress affect cellular pathways and interrupt their functions. Plants respond to these conditions by increasing their antioxidant activities from both enzymatic systems (e.g., superoxide dismutase, catalase, peroxidase, polyphenol oxidase, glutathione S-transferase and ascorbate peroxidase) and non-enzymatic systems (e.g., secondary metabolites, avonoids, terpenoids, phenolic acids, flavonoids, carotenoids, vitamins C and E). In the current study, cowpeas plants cultivated under drought conditions showed a significant increase in their antioxidant activities. ABTS and DPPH have been used to determine the antioxidant activity which can suggest the potential health benefits of crop consumption, consistent with antioxidant compounds (Keshavarz et al. 2016). The change in ABTS and DPPH content and the increase in these compounds in drought conditions might have resulted from oxidative stress in cowpea plants. Moreover, ABTS and DPPH radical scavenging capacity was higher in immature pods than immature seeds or dry seeds because most antioxidant content are found in fresh tissues (Keshavarz et al. 2021; Klunklin and Savage 2017). Also, the results showed that the correlation between antioxidant activity and phenolics compounds was high (data not shown) and ILC482 genotype had the highest ABTS and DPPH value as well as phenolic compounds which could be explained that these variation in plant responses is dependent on genetic factors.

### Leaf nutrients content

Contrary reports showed a reduced uptake of N, P, K, Mg and Ca in legumes (Habibzadeh et al. 2015; McCulloch et al. 2021; Ullah and Farooq 2021) underwater stress which could be explained by the reduction in transpiration rates, altered membrane permeability, decline in the root-absorbing power and impaired nutrient mobility in the soil (Santos et al. 2020).A greater demand of the minerals for seeds can be expected as the plant grows thus, it is expected that lower mineral content of leaves in dry seed stage rather than immature pods. Moreover, leaf nutrients content is affected by developmental stage. Therefore, leaf nutrient status seems to be influenced by age as much as drought stress.

Leaf N content was affected by plant age and water availability (Table 1). As the plant grows, the transfers of N from the various organs of the plant to reproductive parts increases during seed filling (Kurdali et al. 2013).Our results showed that drought stress decreased leaf N content in all development stages and this might be due to lower N uptake and limitation in N fixation. It seems that after reproductive stage, carbon supply which is vital for the survival of *Rhizobia* bacteria was dedicated to developing seeds and pods, therefore the N fixation was reduced (Weidner et al. 2018). Also, it is assumed that the source of carbon was decreased due to lower leaf area index, photosynthesis pigments and photosynthesis capability which resulted to lower assimilation and under drought conditions.

Another reason for leaf N deficit is often associated with decreased nodule mass per plant in stress conditions. In studies that nodule formation has been documented, the effect of unfavorable conditions on nodule mass and N fixation were reported (Mickan et al. 2021). Our results support the idea that the lower leaf N content under drought conditions might be related to carbon supply. It could be concluded that bacteroids might be famished for carbon (malate) and this support the assertion that the impact of drought stress on N fixation and nodule function might be the reason for lower leaf N content under drought condition.

Since water deficit conditions and drying soil hindered water and nutrient flux through the root up to leaves and P is one of the less mobile elements within the soil, its mobility might be decreased by increasing mechanical impendence in water shortage. Also, the preferential allocation of P from shoot area and leaf to root under water scarcity is quite reasonable. The reduction in K concentration of leaf under drought stress could be explained with K as a cheap inorganic osmoticum in turgor process, acting as osmotic adjustment in the root. In agreement with our results, El-Katony et al. (2018) documented that lower K concentration in leaves judged from mobilization of K from shoot area to root for adjustment purpose in swallowwort (*Cynanchum acutum* L.). Also, Goufo et al. (2017) indicated that K contributed to osmotic adjustment in water-stressed cowpeas. The decrease of Mg concentration in leaves under water shortage might be attributed to decreased transpiration rates which has positive correlation with Mg absorption (Chrysargyris et al. 2018). It has been reported that Mg is involved in the regulation of turgor pressure (Wen et al. 2020), stomatal conduction (Rajpoot et al. 2021) and therefore result in water losses regulation. On the other hand, Mg^+^ root content plays a vital role in absorption of NH^4+^ in lettuce seedlings (Zhuet al. 2020). Indeed, Mg^+^ is an important factor in water use efficiency.

## Conclusions

Water stress during the reproductive stage (flowering and pod filling) is the most critical stage which affect the seed yield production and seed quality of cowpea. However, the potential of cowpea to remain as one of the most drought tolerant legumes under climate change threads is encouraging. We found that the water deficit variedly affects biochemical compositions and nutrients concentration. Immature pods had the higher phenolic compounds and antioxidant capacity in all genotypes under the same irrigation regimes. Although subsequent water shortage resulted in higher total phenolics content, the lowest concentration of Mg was achieved in drought conditions. In conclusion, the findings of this study showed that early harvesting of cowpea at immature pod through improving polyphenolic phenolic compounds, better antioxidant capacity and shortage growing season at the same time, making cowpea more suitable for arid and semi-arid regions which may be a helpful strategy to recuperate subsequent drought stress and consequently climate change.

## Data Availability

Data sharing not applicable—no new data generated.
